# Fat-soluble vitamins A and E and health disparities in a cohort of pregnant women at delivery

**DOI:** 10.1017/jns.2018.5

**Published:** 2018-04-12

**Authors:** Corrine Hanson, Marina Verdi Schumacher, Elizabeth Lyden, Dejun Su, Jeremy Furtado, Rex Cammack, Bradley Bereitschaft, Matthew Van Ormer, Howard Needelman, Elizabeth McGinn, Katherine Rilett, Caleb Cave, Rebecca Johnson, Kara Weishaar, Ann Anderson-Berry

**Affiliations:** 1 Medical Nutrition Education, University of Nebraska Medical Center, College of Allied Health Professions, Medical Nutrition Education, Omaha, NE, USA; 2 College of Public Health, University of Nebraska Medical Center, Omaha, NE, USA; 3 Department of Nutrition, Harvard School of Public Health, Cambridge, MA, USA; 4 Department of Geography/Geology, University of Nebraska at Omaha, Omaha, NE, USA; 5 Department of Pediatrics, University of Nebraska Medical Center, 981205 Nebraska Medical Center, Omaha, NE, USA

**Keywords:** Vitamin A, Carotenoids, Food deserts, Food security, Poverty, NHANES, Third National Health and Nutrition Examination Survey, USDA, United States Department of Agriculture

## Abstract

The objective of the present study was to evaluate intakes and serum levels of vitamin A, vitamin E, and related compounds in a cohort of maternal–infant pairs in the Midwestern USA in relation to measures of health disparities. Concentrations of carotenoids and tocopherols in maternal serum were measured using HPLC and measures of socio-economic status, including food security and food desert residence, were obtained in 180 mothers upon admission to a Midwestern Academic Medical Center labour and delivery unit. The Kruskal–Wallis and independent-samples *t* tests were used to compare measures between groups; logistic regression models were used to adjust for relevant confounders. *P* < 0·05 was considered statistically significant. The odds of vitamin A insufficiency/deficiency were 2·17 times higher for non-whites when compared with whites (95 % CI 1·16, 4·05; *P* = 0·01) after adjustment for relevant confounders. Similarly, the odds of being vitamin E deficient were 3·52 times higher for non-whites (95 % CI 1·51, 8·10; *P* = 0·003). Those with public health insurance had lower serum lutein concentrations compared with those with private health insurance (*P* = 0·05), and living in a food desert was associated with lower serum concentrations of β-carotene (*P* = 0·02), after adjustment for confounders. Subjects with low/marginal food security had higher serum levels of lutein and β-cryptoxanthin compared with those with high food security (*P* = 0·004 and 0·02 for lutein and β-cryptoxanthin). Diet quality may be a public health concern in economically disadvantaged populations of industrialised societies leading to nutritional disadvantages as well.

Despite great advances over the past years in agriculture production and nutrition research, millions of people globally are chronically hungry and are affected by several micronutrient deficiencies. This burden of undernutrition can be much more severe among young children and women, especially pregnant and lactating women. Areas with a high prevalence of vitamin A deficiency, for example, often share common dietary and environmental factors, including poverty^(^^1^^)^, infectious diseases^(^^2^^)^ and poor availability of food sources of vitamins.

Fat-soluble vitamins, including vitamins A and E, are essential nutrients throughout the life cycle; however, their influence is particularly critical during periods when cells proliferate rapidly and differentiate, including during pregnancy^(^^1^^)^. Maternal vitamin A deficiency increases the risk of complications during pregnancy and in the postpartum period. It has been positively associated with preterm delivery^(^^1^^)^, maternal infections, night blindness, anaemia^(^^2^^)^, birth defects^(^^3^^)^, and higher morbidity and mortality due to infections^(^^4^^,^^5^^)^, birth weight^(^^5^^)^, diaphragmatic hernia^(^^6^^)^, bronchopulmonary dysplasia and other respiratory diseases^(^^7^^–^^9^^)^. Recent evidence has also shown that vitamin A-related compounds such as carotenes, lutein and lycopene may have unique roles in newborn eye and brain development, independent of retinol^(^^10^^–^^13^^)^. Other biologically significant carotenoids include α-carotene, β-carotene and β-cryptoxanthin, which have pro-vitamin A activity^(^^14^^)^.

Vitamin E is also of critical importance in early infancy, and deficiency at this stage of life has devastating consequences such as intraventricular haemorrhage, bronchopulmonary dysplasia and delays in the development of the central nervous system^(^^15^^)^. Vitamin E occurs in nature in eight different structurally related forms, including four tocopherol forms (α, β, γ and δ) and four tocotrienols (α, β, γ and δ)^(^^16^^)^. α-Tocopherol is quantitatively the major form in humans and has been extensively studied. In contrast, γ-tocopherol, which is the major form of vitamin E in the US diet, has received much less attention^(^^16^^)^. In contrast to the anti-inflammatory properties of the α-tocopherol isoform, the γ-tocopherol isoform has been shown to increase cytokine production (i.e. IL-2) and demonstrate pro-inflammatory properties^(^^17^^–^^21^^)^. Importantly, serum γ-tocopherol isoforms at as little as 10 % of the concentration of α-tocopherol have been shown to ablate the anti-inflammatory benefit of α-tocopherol^(^^19^^)^. As the concentration of γ-tocopherol is high in processed foods^(^^16^^)^, populations with poor diet quality may be consuming large amounts of γ-tocopherol.

Many factors probably contribute to these disparities in dietary quality among Americans, and considerable attention has been focused on income and poor physical access to healthy options^(^^22^^)^. Lower-income individuals consume lower-quality foods, as measured by the Alternate Healthy Eating Index 2010 score in comparison with higher-income individuals. Notably, income disparities in dietary quality widened over the last evaluated years and are also evident when comparing participants in nutrition assistance programmes with non-participants^(^^23^^)^. This finding raises significant concerns that access to nutritional foods is not evenly distributed, and as a result, it is likely that there are populations at risk for food insecurity and nutritional deficiencies in many areas of the country.

Household food insecurity happens when people do not have, at all times, physical, social and economic access to sufficient, safe and nutritious food which meets their dietary needs and food preferences for an active and healthy life^(^^24^^)^. ‘Food desert’ is a term commonly used to describe an area in the USA with limited access to affordable and nutritious food, particularly such an area composed of predominantly lower-income neighbourhoods and communities. Data from a 2009 report showed that 23·5 million people live in low-income areas that are more than 1 mile (1·6 km) from a supermarket or large grocery store^(^^25^^)^.

Despite the potential negative impact, these nutritional deficiencies could have on a developing fetus, nutritional disparities in pregnant women have rarely been examined. Therefore, the objective of the present study was to evaluate serum levels of vitamin A, vitamin E and related compounds in a cohort of pregnant women admitted for delivery in the Midwestern USA in relation to measures of health disparities.

## Materials and methods

### Study characteristics

This was a cross-sectional study evaluating the status of vitamin A, vitamin E and related compounds of 180 pregnant women recruited from the Labor and Delivery unit in a Midwestern United States Academic Medical Center at the time of delivery. Participants were primarily from an urban metropolitan area (91 %); the remainder were from rural areas. A total of sixty pregnant women either did not meet inclusion criteria or declined to participate. This study was conducted according to the guidelines laid down in the Declaration of Helsinki and all procedures involving human subjects were approved by the institutional review board. Written informed consent was obtained from all subjects. Exclusion criteria included congenital abnormalities, gastrointestinal, liver, or kidney disease, or inborn errors of metabolism.

### Evaluation of serum levels

Blood samples were protected from heat and light, promptly processed and frozen at −80°C. Analysis of samples was performed at the Biomarker Research Institute at the Harvard School of Public Health. Measurements of lutein + zeaxanthin, β-cryptoxanthin, lycopene, α-carotene, β-carotene, retinol, α-tocopherol and γ-tocopherol were obtained. Concentrations in plasma samples were measured as described by El-Sohemy *et al.*^(^^26^^)^. Samples were quantitated by HPLC. Because lutein and zeaxanthin co-elute on the chromatogram, the two are grouped and provided as lutein + zeaxanthin. Internal quality control was monitored with four control samples analysed within each run. External quality control was monitored by participation in the standardisation programme for carotenoid analysis from the National Institute of Standards and Technology USA.

Although there is no formal definition of vitamin A status during pregnancy, multiple other studies have defined vitamin A sufficiency during pregnancy as >1·05 µmol/l and deficiency as ≤0·70^(^^1^^,^^27^^–^^30^^)^. Therefore, we used the following retinol categories to classify the vitamin A status of our population: severely deficient, ≤100 µg/l (≤0·35 µmol/l); deficient, >100–200 µg/l (>0·35–0·70 µmol/l); insufficient, >200–300 µg/l (>0·70–1·05 µmol/l); and adequate, >300 µg/l (>1·05 µmol/l). Vitamin E sufficiency was classified as serum levels of α-tocopherol of >8620·7 µg/l (>20 µmol/l) as defined by the Institute of Medicine^(^^14^^)^.

### Evaluation of nutrient intake

The Willett FFQ was administered to all maternal participants at the time of delivery. From responses to the questionnaire, individualised nutrient intake can be calculated based on the known nutrient content of foods. A FFQ has distinct advantages over other methods for assessment of nutrient intake, such as 24 h recalls, as they are reflective of intake over time and can be used to assess intake during the course of the pregnancy. Additionally, many days of recall are required to estimate an individual's dietary intake of vitamin A accurately^(^^31^^,^^32^^)^, making FFQ methodology the preferred method for our objectives. The Willet FFQ has been validated in adults of all ages and sexes and among a variety of socio-economic groups, including populations of low socio-economic status^(^^33^^–^^37^^)^. The FFQ was analysed by trained personnel at the Harvard School of Public Health using the United States Department of Agriculture (USDA) Food Composition Database.

### Food security

Food security was measured using the US Household Food Security Survey Module (US HFSSM) for Measurement of Food Access. The US HFSSM asks respondents to describe behaviours and attitudes that relate to various aspects of the food insecurity experience^(^^38^^)^. Responses to the US HFSSM are summarised in a scale to provide a continuous indicator of the degree of a household's food insecurity. Cut-off points on the scale enable categorical classification of whether households are food secure or not.

### Food deserts

The USDA Economic Research Service publishes a national food desert database at the census-tract level. Census tracts with a significant number or share of people who dwell more than 1 mile (1·6 km) (urban area) or 10 miles (16 km) (rural area) from a supermarket were labelled food deserts. An additional measure accounts for lack of access to transportation.

### Other study variables

Demographic information collected on all maternal participants included age, race, ethnicity, marital status, smoking status and pre-pregnancy BMI, and gestational age of the infant. Race/ethnicity was categorised as Hispanic, non-Hispanic white, non-Hispanic black, and other (including multi-racial). As a surrogate for income, payor type was recorded and classified dichotomously into ‘public’ or ‘private’ health insurance categories.

### Statistical analysis

Descriptive and graphical statistics (means, standard deviations, medians, interquartile ranges, frequencies and percentages) were used to summarise demographic characteristics and clinical measurements of the study population. The prevalence of deficiency and inadequacy in mothers was estimated with proportions and 95 % CI. For the final analysis, the food security variable was dichotomised into ‘food secure (high food security)’ or ‘food insecure (marginal, low, or very low food security)’. The Kruskal–Wallis test was used to compare measures between groups classified by maternal vitamin A concentrations. Independent sample *t*-tests were used to compare continuous measures between dichotomous groups. Linear and logistic regression models were used to adjust associations significant in the univariate analysis for the potential confounders. All nutrient serum and intake values were transformed by taking the natural logarithm (ln) so they would be normally distributed to meet the assumptions of the regression models. Missing values were excluded from the analysis. Potential confounding variables for each analysis were chosen based on significant associations in the univariate models and also with measurements of serum vitamin A-related compounds in the relevant literature. All models of dietary intake included adjustment for energy. A *P* value of <0·05 was considered statistically significant. A sample size of 155 produced a two-sided 95 % CI with a width equal to 0·127 when the sample proportion is 0·50 for the detection of vitamin A insufficiency. This is the widest width that would be produced given a sample size of 155 and possible estimates of vitamin A insufficiency. This same power calculation applies to the race group as well.

## Results

The final number of participants was 180. Mean maternal age was 28·7 (sd 5·6) years; mean maternal pre-pregnancy BMI was 27·1 (sd 6·68) kg/m^2^. In terms of race/ethnic group, 58 % of mothers were white, 14·8 % were African-American, 12 % were Hispanic and 1·6 % Asian/Pacific islander. Most of the mothers were non-smokers (84·13 %), and 14·8 % were current smokers. Baseline characteristics of the maternal cohort are shown in Table 1.

**Table 1.** Sample characteristics, serum concentrations, and dietary intakes (Numbers, mean values and standard deviations, percentages, and medians and ranges)

Maternal characteristic	*n*	Median	Range
Maternal age (years)	180		
Mean		28·7	
sd		5·6	
Maternal BMI (kg/m^2^)	112		
Mean		27·1	
sd		6·6	
Infant gestational age at birth (weeks)	180		
Mean		38·04	
sd		3·1	
Maternal race (%)			
White	111	58·7	
African-American	28	14·8	
Hispanic	24	12·7	
Asian/Pacific Islander	3	1·6	
Other	23	12·7	
Smoking status (%)			
Current smokers	28	15	
Former/never smokers	148	85	
Food security (%)			
High	136	76	
Marginal	17	9	
Low	19	11	
Very low	7	4	
Food desert (%)			
Yes	19	11	
No	148	89	
Health insurance payor status			
Public	103	57·5	
Private	76	42·5	
Maternal serum levels (μg/l)			
Retinol	180	299·5	85·7–734·8
β-Carotene	180	138·2	6·2–1577·4
α-Carotene	180	28·7	3·0–388·4
Lycopene	180	422·0	13·9–1521·7
Lutein + zeaxanthin	180	183·0	26·5–533·0
β-Cryptoxanthin	180	89·6	8·5–356·8
α-Tocopherol	180	12 414·1	175·2–34 687·8
γ-Tocopherol	180	1340·7	151·0–4386·0
Maternal intake (μg/d)			
α-Carotene	132	528·2	6·9–4719·5
β-Carotene	132	4751·0	321·8–17 513·22
β-Cryptoxanthin	132	124·0	11·0–844·5
Lycopene	132	4596·8	170·9–24 654·1
Lutein + zeaxanthin	132	2398·9	177·4–16 316·3
Retinol activity equivalents	132	1325·1	272·3–4724·7
Vitamin E (mg/d)	132	13·7	1·97–221·5

### Serum retinol levels

Overall, the mean serum retinol concentration of the maternal population was 310 µg/l (1·09 µmol/l). Of mothers, 10 % met the criteria for vitamin A deficiency and 41 % were insufficient. Less than half of mothers had adequate serum retinol concentrations (defined as >1·05 µmol/l).

**Fig. 1.**
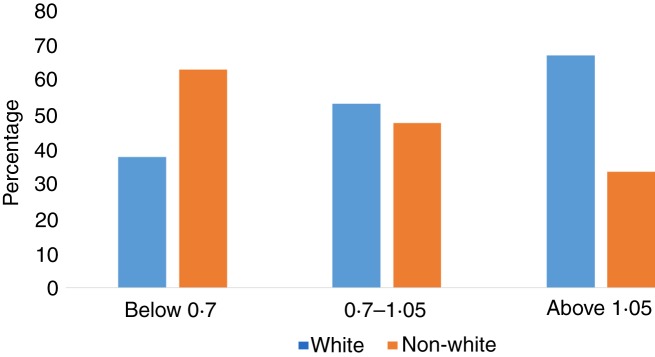
Frequency of vitamin A deficiency, insufficiency and sufficiency by race. The frequency of serum retinol levels <0·70 and ≤1·05 μmol/l was significantly different in non-whites *v*. whites (*P* = 0·04, Fisher's exact test).

The frequency of vitamin A deficiency was significantly different in blacks *v.* whites (*P* = 0·04). Among those with deficient retinol concentrations (≤0·70 µmol/l), 62·5 % of them were non-white. Among the mothers with adequate retinol concentrations (>1·05 µmol/l), 66·7 % were white (Fig. 1). The odds of serum retinol concentrations of <1·05 were 2·17 times higher for blacks when compared with whites (95 % CI 1·16, 4·05; *P* = 0·01) after adjustment for maternal age and smoking status. Results of the logistic regression model for vitamin A insufficiency are shown in Table 2.

**Table 2.** Results of the logistic regression analysis for vitamin A deficiency (serum retinol ≤1·05 μmol/l) (Odds ratios and 95 % confidence intervals)

Variable	OR	95 % CI	*P*
Race (non-white *v*. white)	2·17	1·16, 4·05	0·01
Maternal age	1·01	0·96, 1·07	0·61
Maternal smoking status	0·69	0·29, 1·63	0·39

### Serum levels of vitamin A-related compounds

In the univariate analysis, a statistically significant association was seen between race and maternal serum lycopene. Mean serum levels of total lycopene were significantly higher in white mothers compared with non-white mothers (498·8 *v.* 419·2 μg/l for white *v.* non-white, respectively; *P* = 0·03). Those with private insurance had higher levels of lutein (220·6 *v.* 184·3 μg/l; *P* = 0·003) and β-carotene (282·7 *v.* 171·4 μg/l; *P* = 0·01) when compared with those with public insurance. Living in a food desert was associated with lower levels of β-cryptoxanthin (100·7 *v.* 107·4 μg/l; *P* = 0·02), lycopene (399·1 *v*. 475·8 μg/l; *P* = 0·04) and β-carotene (143·5 *v.* 228·5 μg/l; *P* = 0·005). In contrast with these findings, those with low food security had serum lutein levels that were higher than those who were food secure (221·1 *v.* 192·3 μg/l; *P* = 0·04 for low and high food security, respectively). After adjusting for age, smoking status, race, food desert and food security, insurance status maintained a significant association with serum lutein (*P* = 0·004), and β-carotene was inversely associated with living in a food desert (*P* = 0·02). In contrast to our hypothesis, those with low/marginal food security had higher levels of lutein and β-cryptoxanthin compared with those with high food security (*P* = 0·004 and 0·02 for lutein and β-cryptoxanthin, respectively).

### Serum levels of vitamin E tocopherols

**Fig. 2.**
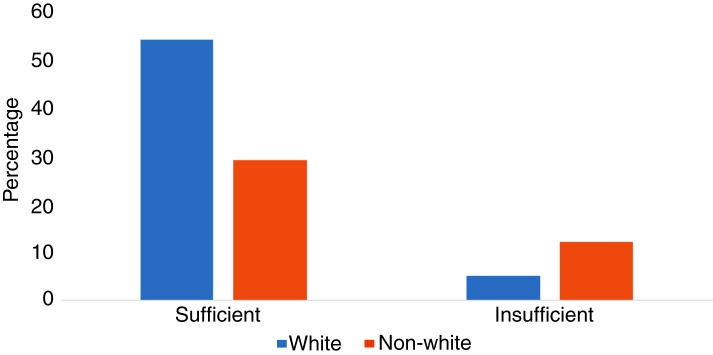
Frequency of vitamin E insufficiency and sufficiency by race. The frequency of serum α-tocopherol levels ≤8620·7 µg/l (<20 µmol/l) was significantly different in non-whites *v*. whites (*P* = 0·004, Fisher's exact test).

When classified by vitamin E-‘sufficient’ status (serum α-tocopherol levels of >8620·7 µg/l), 64 % of white mothers were sufficient, compared with only 36 % of non-white mothers (*P* = 0·004) (Fig. 2). After adjustment for confounders (maternal age and smoking status), the odds of being vitamin E insufficient were 3·52 times higher for non-whites when compared with whites (95 % CI 1·51, 8·10; *P* = 0·003). The results of the logistic regression models for vitamin E sufficiency are shown in Table 3.

**Table 3.** Results of the logistic regression analysis for vitamin E insufficiency (serum α-tocopherol levels of ≤8620·7 µg/l) (Odds ratios and 95 % confidence intervals)

Variable	OR	95 % CI	*P*
Race (non-white *v*. white)	3·52	1·51, 8·10	0·003
Maternal age	0·97	0·90, 1·05	0·47
Maternal smoking status	1·82	0·66, 5·05	0·25

In the univariate analysis, mothers with public insurance had higher levels of γ-tocopherol when compared with mothers with private insurance (1516·6 *v.* 1345·4 µg/l; *P* = 0·003). After adjustment for age, race, smoking, food security and food desert status, this relationship was no longer significant (*P* = 0·10).

### Dietary intake

Out of 180 maternal participants, 132 completed the FFQ. In the univariate analysis, intakes of β-carotene and lutein differed by payor source, with intakes of both compounds being higher in those with private insurances compared with those with public insurance (5888·3 *v.* 4923·9 µg/d, *P* = 0·04, and 3439·8 *v.* 2687·9 µg/d, *P* = 0·04, for β-carotene and lutein, respectively). After adjustment for energy, race, smoking and food security, these relationships were no longer significant.

The proportion of mothers who met the RDA for vitamin E as α-tocopherol (15 mg/d)^(^^14^^)^ was also significantly different between food security groups, with 47 % of mothers with high food security meeting the RDA for vitamin E compared with only 9 % of mothers with marginal or low food security (*P* = 0·02). After adjustment for confounding factors including energy, race, insurance, maternal age and food desert status, those with marginal or low food security had 5·4 times higher odds of intakes below the RDA for vitamin E when compared with those with high food security (95 % CI 1·73, 17·0; *P* = 0·003). The results of the logistic regression model for vitamin E intake below the RDA is shown in Table 4.

**Table 4.** Results of the logistic regression analysis for vitamin E intakes below the RDA (15 mg α-tocopherol/d) (Odds ratios and 95 % confidence intervals)

Variable	OR	95 % CI	*P*
Household food security (low *v*. high)	5·55	1·72, 17·27	0·003
Maternal age	0·94	0·86, 1·02	0·11
Energy intake	0·99	0·98, 1·00	0·0005
Insurance status	1·13	0·47, 2·74	0·78
Food desert status	1·61	0·37, 7·0	0·52

Consistent with our hypothesis that intakes of γ-tocopherol would be higher in groups of low socio-economic status, those with high food security in our cohort took in 9·1 mg γ-tocopherol/d, while those with low or marginal food security took in 11·3 mg/d (*P* = 0·04). This relationship was somewhat attenuated after adjustment for confounding factors of age, smoking, race, food security, food desert status, payor type and energy (*P* = 0·09).

## Discussion

Our study finds that race and insurance status were associated with both serum levels and intakes of vitamin A, vitamin E and related compounds. Non-white women in our cohort were at significantly higher risk for deficiencies in both vitamin A and vitamin E, and those women of lower socio-economic status had lower levels of serum lutein and β-carotene, and higher intakes of γ-tocopherol. Given the importance of fetal exposure to these compounds during pregnancy, these results point to the profound impacts of nutritional disparities in populations who already suffer from high rates of disparate maternal–child outcomes.

Recently, concerns have been raised about the diet quality of susceptible populations, including women of childbearing age and pregnant women, and several studies have implicated poverty and race as determinants of nutritional inequities. A study using data from the Third National Health and Nutrition Examination Survey (NHANES) demonstrated that women of childbearing age in the USA are not consuming adequate amounts of multiple micronutrients, and this was especially true for women in minority ethnic groups, as well as women of lower socio-economic status^(^^39^^–^^41^^)^. Other studies have shown that lower-income households and racial minorities have less access to, and are less likely to consume, high-quality foods such as fruits and vegetables^(^^42^^–^^44^^)^, the primary sources of pro-vitamin A compounds, including lycopene. Only 19 % of Hispanics and 22 % of African Americans consume vegetables ≥3 times per d, the lowest of any US racial or ethnic group^(42)^. Results from NHANES III (1999–2002) show that non-Hispanic blacks were 43 % less likely and Hispanics 5 % less likely than whites to meet USDA fruit and vegetable guidelines^(^^45^^)^.

Our results found that non-white women were more likely than white women to be deficient or insufficient in vitamin A. Racial differences in serum retinol levels have previously been shown using older NHANES data, with non-Hispanic black and Mexican American females being more likely than non-Hispanic white females to have low serum retinol concentrations^(^^46^^,^^47^^)^. A previous study by our group used more recent NHANES data to assess serum retinol concentrations from 3170 women of childbearing age (14–45 years of age). Those results indicate that poverty score and race were significantly correlated with vitamin A status after adjusting for confounding variables like inflammation, oestrogen use and smoking status. Retinol concentrations of <1·05 µmol/l were 1·85 times higher for those of lower socio-economic status when compared with women of higher status. Non-Hispanic black and Mexican American females are more likely than non-Hispanic white females to have low serum retinol concentrations. Dietary vitamin A intake was statistically significantly lower in groups with higher poverty scores (*P* = 0·004); women of lower socio-economic status took in an average of 93·9 less retinol activity equivalents when compared with women of higher status^(^^48^^)^. There is other limited survey information in US populations that also suggests that those from poorer neighbourhoods and with evidence of lower-quality dietary intakes in the USA have a higher prevalence of lower serum retinol values^(^^49^^,^^50^^)^.

We also investigated the impact of other socio-economic indicators on vitamin A and E status that have not been well explored, including food insecurity. Food insecurity happens when people do not have, at all times, physical, social and economic access to sufficient, safe and nutritious food which meets their dietary needs and food preferences for an active and healthy life^(^^24^^)^. We found that pregnant women with food insecurity tended to have intakes of γ-tocopherol that were higher than women who were food secure. In the US diet, γ-tocopherol is the principal vitamin E isoform consumed, being about 2·5 times as abundant in food as α-tocopherol. The main sources of γ-tocopherol include soyabean, maize and peanut oils, making it abundant in both fast foods and highly processed foods. The average plasma γ-tocopherol level in the USA has been reported as approximately 2–6 times higher than that reported for six European countries that consume higher amounts of α-tocopherol-rich foods^(^^51^^)^. It is presumed that as countries assume Western lifestyles, diets change including increased consumption of soyabean oil with corresponding increases in plasma γ-tocopherol. The impact of these increasing levels of serum γ-tocopherol on maternal–child outcomes is not yet well understood. In one case–control study of 1605 pregnant women in Bangladesh, a low γ-tocopherol concentration was associated with a decreased risk of miscarriage (OR 0·62; 95 % CI 0·41, 0·93)^(^^52^^)^; however, more research is required to assess the impact and optimal ratio of these vitamin E tocopherol compounds.

Food insecurity often affects young children and women, especially pregnant and lactating women, most in low- and low/middle-income countries^(^^53^^)^; however, food insecurity remains a problem for low-income families in the USA^(^^22^^)^. The prevalence of household food insecurity in the USA appears to be double that observed in Canada. In 2012, the most recent year for which comparable data are available, the problem affected 7 % of Canadian households and 15 % of US households^(^^54^^)^. A recent study compared data from two nationally representative surveys, the 2004 Canadian Community Health Survey and the 2003–2006 NHANES, to estimate prevalence of inadequate intakes of vitamins A and C, folate, Ca, Mg and Zn among youth and adults among food-secure and food-insecure households. Surprisingly, larger gaps in the prevalence of inadequate intakes between those in food-secure and food-insecure households were observed in Canada than in the USA. One salient difference relevant to food insecurity is the existence of extensive public food programmes in place in the USA to provide assistance to vulnerable households^(^^23^^,^^54^^)^. This may, in part, explain the finding in our study showing that lower levels of food security were associated with increased serum levels of both lutein and β-cryptoxanthin. It is possible that such programmes have an impact on the food-insecure individuals in our study, increasing their access to foods high in compounds such as lutein.

Our study is one of the first to examine the impact of food deserts on the fat-soluble vitamin status during pregnancy. ‘Food deserts’, commonly used to describe communities with little or no access to healthy food, currently affect millions of Americans: the USDA reports that about 23·5 million people currently live in food deserts, including 6·5 million children^(^^55^^)^. Recently, the USDA, and the Departments of Health and Human Services and the Treasury joined together to develop the Healthy Food Financing Initiative (HFFI) to improve access to healthy, affordable foods in food deserts^(^^23^^)^. We did find significant associations between food desert status and vitamins A and E in the univariate analysis; after adjusting for confounders, serum β-carotene levels remained significantly lower in those participants who live in a food desert. This finding may not be surprising, as serum carotene levels are considered biomarkers of fruit and vegetable intakes, the very types of foods which are lacking in food deserts^(^^14^^)^. Although these types of deficiencies have been classically associated with low- and middle-income countries, the micronutrient deficiencies seen in our study may be a reflection of people living under poor conditions in the country's metropolitan areas. It also reflects the lifestyle changes occurring in urban areas, where modernisation leads to greater consumption of industrialised non-nutritious foods.

### Conclusion

Our results demonstrate that discrepancies in vitamin A and E status are a concern in nutritionally disadvantaged populations of industrialised societies. More research is needed to determine what type of interventions will be best for specific populations, especially the low-income and minority populations that bear a greater burden of vitamin A and E inadequacy. As the population of the USA becomes more diverse^(^^56^^)^, issues regarding health and disparities in diet become even more salient.
